# Degradation Rate/Vulnerability Potential and Fertility Status of Luvisols in the Mandara Mountains (Far-North Cameroon)

**DOI:** 10.1155/2024/6565723

**Published:** 2024-05-06

**Authors:** Estelle Lionelle Tamto Mamdem, Désiré Tsozué, Emmanuel Matakon, Michael Roi Apiniel Atourakail, Nérine Mabelle Moudjie Noubissie, Simon Djakba Basga, Aubin Nzeugang Nzeukou, Dieudonné Lucien Oyono Bitom

**Affiliations:** ^1^Department of Earth Sciences, Faculty of Sciences, University of Yaoundé 1, P.O. Box 812, Yaoundé, Cameroon; ^2^Department of Earth Sciences, Faculty of Science, University of Maroua, P.O. Box 814, Maroua, Cameroon; ^3^Institute of Agricultural Research for Development (IRAD), P.O. Box 415, Garoua, Cameroon; ^4^Department of Soil Science, Faculty of Agronomy and Agricultural Sciences, University of Dschang, P.O. Box 222, Dschang, Cameroon

## Abstract

Soil degradation emerges as one of the major problems in the locality of Sir in the Mandara Mountains, Far-North Cameroon. Inappropriate agricultural techniques resulting in land use change affect soil functions and seriously harm forest ecosystems. This study was conducted to analyse the current character of soils and access their degradation and their level of fertility. Twenty soil samples were taken at 15 cm depth. This includes ten in the plot under forest reserve and ten others in the plot under cultivation. Cultivation is responsible for the increase in bulk density (BD) (1.59 to 2.23 g/cm^3^), Mg (4.76 to 6.40 cmol·kg^−1^), Ca (10.44 to 11.26 cmol·kg^−1^), P (7.93 to 9.93 g/kg), and Mg/K (2.28 to 5.84) and decrease in CEC (38 0.15 to 31.46 cmol·kg^−1^), OM (2.76 to 1.08%), OC (1.66 to 0.62%), total nitrogen (0.08 to 0.05%), K (4.59 to 1.15 cmol·kg^−1^), Na (1.32 to 0.91 cmol·kg^−1^), C/N (25.69 to 13.86), and Ca/Mg (2.32 to 1.89). This variability in physicochemical properties reflects progressive soil degradation. Cultivated soils are subject to severe degradation or potential vulnerability (SDR/Vp = 4/2) due to texture, organic carbon, soil aggregate stability, sealing index, and total nitrogen. On the other hand, soils under forest reserve are subject to severe degradation or vulnerability due to the total nitrogen and sodium absorption ratio. The soils of the study area are subject to severe and extreme potential degradation or vulnerability due to BD, respectively, under forest reserve and cultivation. Two classes of fertility were identified: class II (plots under forest reserve) having a good level of fertility, characterized by good physical properties and severe limitations in nitrogen and phosphorus and class IV (cultivated plots) with a low level of fertility due to severe limitations in organic matter, phosphorus, and poor physical characteristics. The best indicator of the good quality of the luvisols of Sir is the pH, while the bulk density is an indicator of severe to very extreme degradation or high to very high vulnerability. The application of organic and mineral amendments is essential for raising the organic matter and nitrogen and phosphorus contents in these soils.

## 1. Introduction

Population growth in sub-Saharan Africa has led to an increase in food demand [1]. Soil fertility management is fundamental for sustainable agriculture ensuring food security and the standard of living of the population [2, 3]. However, increasing agricultural production requires good knowledge of the concept of soil degradation and fertility [4, 5]. Intensive agriculture and the search for new fertile lands result in pressure on the ecosystem, which, in turn, exposes the soil to degradation issues and considerably reduces its fertility [6]. Similarly, poor land use and management expose agricultural soils to the risk of degradation.

Like most other areas in the world, the Sudano-Sahelian zone of Africa is suffering from the degradation of its natural resources [7]. In Cameroon, particularly in the Far-North region, degradation emerges as one of the major problems in the area, due to the impact of human activity on their dynamics [8]. Ignorance of soil heritage, resulting in the change in land use, mainly the passage from forest land to agricultural land, is very frequent [9]. This practice affects soil functions and seriously harms forest ecosystems, especially since agriculture is identified as being by far the main cause of tropical deforestation [10]. The degraded soils, with low production potential, when they are not abandoned, no longer allow satisfactory agricultural production, thus exposing the population to malnutrition, starvation, and food insecurity [2]. Under these conditions, agro-socio-economic sustainability seems inaccessible and the protection of the environment and natural resources cannot become the major concern of farmers, which is mainly because of their very short-term future [11]. It seems necessary and imperative to rethink the management and restoration of agricultural land [12, 13] and in particular those from the Sudano-Sahelian zone whose crops contribute directly to the feeding of the population [8]. In addition to erosion, identified by far as the main factor of soil degradation in the Sudano-Sahelian zone [14], overgrazing and the uncontrolled use of pesticides/chemical fertilizers are also other factors responsible for soil degradation [8, 15, 16].

Inappropriate agricultural techniques resulting in land use change as a consequence of human activities affect soil functions [8, 9, 14, 15]. This leads to soil fertility degradation problems and seriously harms forest ecosystems. This is reflected in the Far-North region of Cameroon and more particularly in the Mandara Mountains by the reduction in yields of the main crops [14]. There is an increase in the need for information likely to improve agricultural production in this part of the country. The decrease in yields mainly affects crops which directly contribute to feeding populations and therefore to food security. The objective of the present study is to analyse the current character of soils and access their degradation rate/vulnerability potential and level of fertility. To do this, the locality of Sir in the Mandara Mountains was chosen to conduct the study. The analysis was based on the comparison of the physicochemical characteristics of soils in two contrasted plots, one is a forest reserve (control) and the other is under cultivation, with different management systems. In medium-dated, the results of this study will serve in the development of strategies for the management and restoration of agricultural land, thus promoting an increase in agricultural production in the Sudano-Sahelian zone.

## 2. Materials and Methods

### 2.1. Study Area

**Figure 1:**
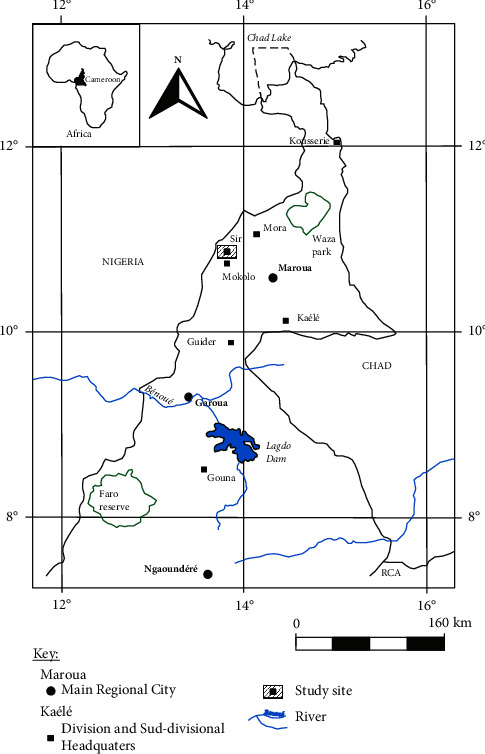
Location of the study area.

The study area was located in the Mandara Mountains, precisely at Sir in the Kapsiki plateau (Figure 1) in the Mayo-Tsanaga Division, between latitudes 10°22′–10°44′ North and longitudes 13°31′–13°47′ East. This Kapsiki Plateau represents the northernmost volcanic zone of the Cameroon Volcanic Line [17]. It was an asymmetrical horst that covered an area of 150 km^2^, located along the Cameroon-Nigeria border. This locality was influenced by a Sudano-Sahelian mountain type climate [18], with a long dry season from October to June and a short rainy season from July to September, with a mean annual rainfall and temperature of 1074 mm and 26°C, respectively [19]. The geologic substratum consisted mainly of gneisses, migmatites, and granites [20–22]. Locally, it was covered by Lower Cretaceous continental sediments cut by trachytic and rhyolitic dykes and numerous basaltic dykes and flows [20].

### 2.2. Experimental Plots

The study was carried out in two plots, including one under natural forest reserve (control) and another under cultivation. The current surface state of each plot of about 9 km^2^ is shown in Table 1. Different land management practices included ploughing, removal of crop residues, uncontrolled use of chemicals products (fertilizers and pesticides), application of organic manure, fallow, mulching, crop rotation, crop association, weeding, ridging, burning and direct seeding in the cultivated plot, as well as increased grazing in forest reserves. These different modes of land use have already been reported by several authors [7, 8, 23]. The main crops grown in the cultivated plot were millet (Pennisetum glaucum), sorrel (Rumex acetosa), maize (Zea mays), Bambara groundnut (Vigna subterranea), cowpea (Vigna unguiculata), sesame (Sesamum indicum), potato (Ipomoea batatas), rice (Oryza sativa), and soybean (Glycine max) (Table 1). The forest reserve, in addition to grazing, was subjected to uncontrolled cutting of trees as a consequence of the growing needs of wood by expanding urban populations, as already noted in the Zamai savannah by Tsozué et al. [23]. There was an important biological activity, characterized by the presence of many termite mounds.

**Figure 2:**
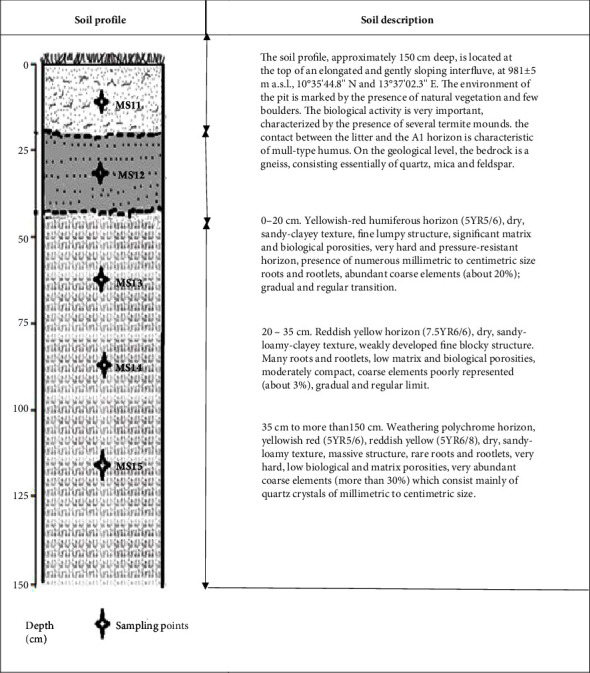
Macroscopic organization of the soil in the study area.

**Figure 3:**
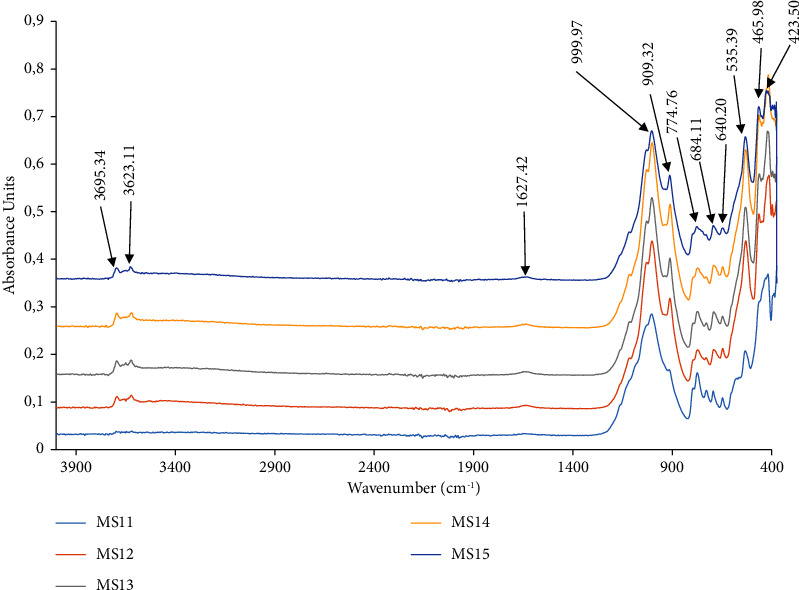
Infrared spectra of the studied soil.

The studied soil profile has a thickness of 1.5 m (Figure 2). It is composed of three horizons which are, from top to bottom, a yellowish-red humiferous horizon with sandy-clayey texture and fine lumpy structure, a reddish-yellow horizon with loamy-clayey texture and weakly developed blocky structure, and a weathering polychrome horizon with sandy-loamy texture and massive structure. Mineralogically, it was constituted of kaolinite (3695.34 cm^−1^, 3623.10 cm^−1^, 909.31 cm^−1^), quartz (999.96 cm^−1^, 774.76 cm^−1^, 684.11 cm^−1^, 465.98 cm^−1^, 423.49 cm^−1^), smectites (1627.42 cm^−1^), goethite (640 cm^−1^, 684 cm^−1^), and sepiolite (535.39 cm^−1^) (Figure 3). It was marked by a subsurface horizon (50 to 75 cm) with higher clay content than the overlying horizon. This horizon was characterized by a fine to massive blocky structure, with a sandy clay-loam texture, high chroma, and a reddish-yellow colour (5 YR 6/8). Cation exchange capacity and base saturation rate in this horizon were high. All of these observed properties were characteristics of an argic horizon. Referring to the World Reference Base for Soil Resources, these properties were those of luvisols. No particular character was noted along the soil profile, which allowed them to be classified as haplic luvisols. Throughout the soil profile, cation exchange capacity was generally high due to the strong activities of the 2/1 type clays, in particular those of montmorillonites and sepiolites (35.74 ± 13.18 cmol·kg^−1^; CV = 37%). Base saturation was moderate (51.49 ± 19.53%; CV = 38%) (Table 2). The studied soil thus belonged to the epidystric haplic luvisol groups.

### 2.3. Soil Sampling and Laboratory Analysis

Fieldwork took place in two phases. The first phase consisted of direct observations, description of environmental settings, and soil survey in order to choose the position of drills and pits. Within each plot, seven transects along which a total of 56 drills of 1.20 m deep were performed to appreciate the morphological organization of soils were opened. This permits us to note that the studied soils are homogenous, leading to the choice of the digging point for the representative soil profile in the forest reserve. A representative soil profile was thereafter dug in the forest reserve and described in detail according to the standard procedure, for soil characterization and classification. The main search characters were colour, thickness of horizons, coarse elements, texture, structure, porosity, roots and roolets, consistency, and boundaries between horizons. The Munsell Soil Colour Chart was used for colour appreciation. Secondly, due to the availability of nutrients and microbiota found mainly in the most superficial layers [23], surface soil samples (0–15 cm) were taken from the forest reserve and cultivated plot. At each sampling point, approximately 1 kg of soil sample was taken. Three repetitions were carried out in the field, which led to the collection of 40 samples per plot. These samples subsequently underwent quartering operations. A total of twenty composite samples were taken, including ten from the forest reserve and ten others from the cultivated plot. The collected soils were placed in polythene bags and labelled for later laboratory analyses. The history and different land management practices of the cultivated plot were obtained from direct interviews with the farmers. The samples were air-dried at room temperature for approximately one week and passed through a 2 mm sieve for physicochemical analyses in the laboratory. Methods used for physicochemical analyses were those already used by Tsozué et al. [23, 24] and Issine et al. [25]. For soil texture analysis, soil organic matter and carbonates were removed with hydrogen peroxide (30%) and diluted hydrochloric acid (10%), respectively. Sand was separated from silt and clay by wet sieving. Then, silt and clay were dispersed with sodium hexametaphosphate, and particle size distribution was analysed using the pipette method. Clay and fine silt were separated by wet sieving, and coarse silt was estimated by subtraction [26]. Soil pH was determined using a glass electrode in a 1 : 2.5 soil : water suspension for pH_H2O_ and a 1 : 1 soil : KCl (1 M) suspension for pHKCl, using a soil-to-water ratio extracts of 1 g : 2.5 ml [27]. Bulk density (BD) was measured by the paraffin-coated clod method [28]. Field-moist clod samples were carefully collected at natural breaks to form smaller clods. Each clod was dried in an oven at 105°C for at least 48 h and weighed. After tying thread around the clods, they were repeatedly dipped in melted paraffin until air bubbles were no longer observed to provide a thin waterproof coat. The volume of the clods was determined by the displacement of water in graduated cylinders. Exchangeable cations were extracted by a solution of ammonium acetate (1 N NH_4_OAc) at pH 7, and their concentrations were determined by atomic absorption spectrometry for Ca and Mg and by flame emission spectrometry for K and Na. Cation exchange capacity (CEC) was also determined using the ammonium acetate method at pH 7, by a direct continuation using a 1 N potassium chloride (KCl) saturation solution [29]. Base saturation (V) was determined according to the formula (SBE/CEC) × 100. Total nitrogen was obtained after heat treatment of each sample in a mixture of concentrated sulfuric acid and salicylic acid. The mineralization was accelerated by a catalyst consisting of iron sulfate, selenium, and potassium sulfate. The mineralization was followed by distillation via conversion of nitrogen into steam in the form of ammonia (NH_3_), after alkalinization of mineralized extract with NaOH. The distillate was fixed in boric acid (H_3_BO_3_) and then titrated with sulfuric acid or diluted hydrochloric acid (0.01 N). Organic carbon (OC) content was determined by wet oxidation according to Walkley and Black [30] using a mixture of sodium dichromate and concentrated H_2_SO_4_. The organic matter (OM) content was calculated from the formula OM = 1.724 × OC and then the C/N ratio. The available phosphorous content was analysed using the Bray II method, which combines extraction of phosphorus in the acid medium and their complexation with ammonium fluoride (NH_4_F). The quantity of available phosphorus was obtained by spectrophotometry in the presence of blue molybdenum (MoO_3_) [31]. For mineralogical analysis, diffuse reflectance infrared spectra were recorded between 4000 and 400 cm^−1^, using a FTIR Perkin Elmer 2000 spectrometer (Perkin Elmer, Waltham, MA, USA) equipped with a deuterated triglycine sulfate (DTGS) detector. Air-dried samples were analysed at room temperature using diamond attenuated total reflectance (ATR) accessories (Perkin Elmer). The spectrum resolution was 4 cm^−1^, and the accumulation time was 5 min.

### 2.4. Calculation of Soil Degradation Rate/Vulnerability Potential and Fertility Parameters

The physicochemical properties commonly used are OC, OM, N, C/N, available P, Ca, Mg, K, Na, SBE (sum of exchangeable bases), CEC, textural class, BD, and pH_H2O_. These soil properties are used to calculate soil degradation rate/vulnerability potential (SDR/Vp) and soil fertility parameters such as Forestier index (IF), soil aggregate stability index (ISS), soil sealing index (IB), and sodium absorption ratio (SAR).

For the SDR, the weighting sequence was as follows: 1 = none, 2 = slight, 3 = moderate, 4 = severe, and 5 = extreme. In this way, good soils have the lowest SDR and poor soils the highest value. For the Vp, the weighting was the reverse as follows: 5 = very low, 4 = low, 3 = medium, 2 = high, and 1 = very high [4, 12, 28]. The cumulative rating index corresponds to the sum of different SDR/Vp weighting values [28].

The soil aggregate stability index (ISS) related to soil resistance to disturbance from external forces was evaluated using the formula of Pieri [32]. It is in fact a measurement of the resistance of the aggregates, therefore of the structural porosities, against agents that can destroy them and in particular against water. This formula is defined as follows:

(1) $$\begin{array}{c} \text{ISS }\left(\%\right)=\frac{1.724\;x\text{ OC}}{\left(L+A\right)}x100, \end{array}$$

with OC as the soil organic carbon, *L* as the silt fraction, and *A* as the clay fraction. An ISS > 9% indicates stable structure, 7% < ISS ≤ 9% indicates a low risk of structural degradation, 5% < ISS ≤ 7% indicates a high risk of degradation, and ISS ≤ 5% indicates structurally degraded soil.

The soil sealing or impermeabilization index (IB) related to the risk of soil erosion and compaction was estimated using the Remy formula [33]:

(2) $$\begin{array}{c} \text{IB }\left(\%\right)=\frac{\left(1.5\text{xLf}\right)+\left(0.75\text{xLg}\right)}{A-10\text{xOM}}-C, \end{array}$$

with *C* being equal to 0.2 × (pH-7); Lf, the fine silt; Lg, the coarse silt; *A*, the clay; and OM, soil organic matter content. An IB < 1.4 indicates soils without a risk of thrust and without a risk of erosion; 1.4 < IB ≤ 1.6 indicates soils with a low risk of erosion; 1.6 < IB ≤ 1.8 indicates soils with a medium risk of erosion; IB ≥ 1.8 indicates soils with a high risk of erosion.

The Forestier index (IF) was assessed using the following formula [34]:

(3) $$\begin{array}{c} \text{IF}=\frac{{\text{SBE}}^{2}}{\left(A+\text{Lf}\right)}, \end{array}$$

with SBE, the sum of exchangeable cations; *A*, the clay fraction; and Lf, the fine silt fraction. An IF < 1.5 indicates soils with low nutrient reserves, and an IF > 1.5 indicates soils with good nutrient reserves.

The sodium absorption ratio (SAR) was calculated by the following relationship [28]:

(4) $$\begin{array}{c} \text{SAR}=\frac{\left[\text{Na}+\right]}{\sqrt{\left[\text{Ca}2+\right]+\left[\text{Mg}2+\right]}}x100. \end{array}$$

SAR < 10 indicates soils with no limitation; 10 < SAR < 12 indicates soils with slight limitation; 12 < SAR < 15 indicates moderate limitation; 15 < SAR < 20 indicates severe limitation; SAR > 20 indicates extreme limitation.

Fertility classes were obtained based on the criteria for evaluating soil fertility classes of Quemada and Cabrera [35], modified by Nguemezi et al. [5]. Class I: soil characteristics are not present or present only weak limitations; Class II: soil characteristics do not have more than three moderate limitations possibly associated with low limitations; Class III: soil characteristics have more than three moderate limitations possibly associated with a single severe limitation; Class IV: soil characteristics have more than one severe limitation.

### 2.5. Statistical Analysis

To assess the main characteristics of the sampled population, the various physical and chemical properties of soils were subjected to standard statistical analyses (mean, maximum-minimum values, standard deviation, and the coefficient of variation). The normality of the distribution was studied by the Anderson–Darling normality test. The significance test was applied to the average values of the physicochemical properties in order to measure the variability of the parameters between the two plots, soil under forest reserve and soil under cultivation. Subsequently, correlation analyses (Spearman test) on the different variables were also carried out to measure the links between them. Finally, to evaluate the effect of cultivation on the different properties and parameters of the soil, principal component analysis (PCA) was performed. Significance was considered at *p* < 0.05. All statistical analyses were performed using XLSTAT software for Excel (version 2014.5.03).

## 3. Results

### 3.1. Physicochemical Characteristics of Soils

Sand represents the important particle size fraction both in the forest reserve and in the cultivated plot. Its proportions are between 60.6 and 80.6%, with an average of 73.88 ± 5.89% in the forest reserve and between 68 and 86%, with an average of 77.2 ± 4.52% in the cultivated plot (Table 3). Silt was the second largest grain size fraction in both plots. Its contents vary between 12 and 26%, with an average of 17.67 ± 4.34% in the forest reserve and between 7.6 and 18.6%, with an average of 13.6 ± 2.98% in the cultivated plot. As for clay, its average contents are 8.45 ± 2.07% and 9.2 ± 2.49%, respectively, in the forest reserve and in the cultivated plot. In the cultivated plot, there is a drop in silt content, while the sand and clay contents increase compared to the control (Table 4). Sand and clay contents did not significantly differ between the two plots, unlike silt content which differed significantly (*p* = 0.034) (Table 5). The bulk density (BD) of the plots under forest reserve has an average of 1.59 ± 0.37 g/cm^3^, ranging between 0.79 and 2.11 g/cm^3^, while that of the cultivated plots varies from 1.49 to 3.44 g/cm^3^, with an average of 2.23 ± 0.71 g/cm^3^ (Table 4). Soil BD increases with cultivation but does not differ significantly between the two plots (*p* = 0.256) (Table 5). The soil aggregate stability index (ISS) of the studied soils differed significantly from one plot to another (*p* = 0.002) (Table 5). Its values calculated in soils under forest reserve are between 2.66 and 17.59%, with an average of 10.48 ± 4.02% (Table 3). In the cultivated plots, the values vary between 0.84 and 8.71%, with an average of 4.62 ± 2.93% (Table 4).

The pH values are similar and do not show any significant difference from one plot to another. The studied soils are slightly acidic, with an average pH value of 6.4 ± 0.5 in the forest reserve and 6.3 ± 0.55 in the cultivated soils (Tables 3 and 5). The Mg content is high in the reserve and very high in the cultivated soils. Ca has a high content in both cases. For Mg, the levels in the forest reserve evolve unevenly and are between 2.48 and 6.56 cmol·kg^−1^ of soil, with an average of 4.76 ± 1.39 cmol·kg^−1^ (Table 3). In the cultivated plot, its values oscillate between 3.12 and 9.36 cmol·kg^−1^, with an average of 6.40 ± 1.92 cmol·kg^−1^ (Table 5). As for the Ca, the average content in the forest reserve is 10.41 ± 2.01 cmol·kg^−1^ and it varies between 7.68 and 13.52 cmol·kg^−1^ (Table 3). For the cultivated plots, they vary between 7.76 and 16.56 cmol·kg^−1^, with an average of 11.26 ± 2.5 cmol·kg^−1^ (Table 5). The Mg and Ca contents showed no statistically significant difference between the two plots (Table 4). The soils under forest reserve have a high content of Na and very high content of K with an average content of 4.59 ± 3.45 cmol·kg^−1^ and 1.32 ± 0.65 cmol·kg^−1^, respectively. In the cultivated plot, the average K content is 1.15 ± 0.29 cmol·kg^−1^ and that of sodium is 0.91 ± 0.11 cmol·kg^−1^ (Table 5). The average K and Na contents in the soils under the forest reserve are higher than those of the cultivated plots (Table 4). The cation exchange capacity of soils under reserve is high. Its values are between 25.44 and 71.44 cmol·kg^−1^, with an average of 38.15 ± 12.39 cmol·kg^−1^ against an average of 31.46 ± 12.80 cmol·kg^−1^ in cultivated soils (Table 5). These average contents vary significantly between the two plots (*p*=0.023) (Table 4). Base saturation (V) is higher in cultivated plots than in forest reserve plots. The average values are 67.14 ± 16.38% in the cultivated soils and 57.07 ± 15.87% in the soils under reserve (Tables 3 and 5). The OM content is moderate in the forest reserve, while it is low in the cultivated plots. Its values oscillate between 0.59 and 4.63%, with an average of 2.76 ± 1.2% in the reserve (Table 3). In the cultivated plot, the OM content varies between 0.17 and 2.09%, corresponding to an average of 1.08 ± 0.70% (Table 5). A significant difference in OM content was noted between the two plots (*p*=0.001) (Table 4). With regard to nitrogen, lower average concentrations were measured in the cultivated plot, while in the reserve, the concentrations were higher. The average concentrations obtained are 0.08 ± 0.03% for the reserve and 0.05 ± 0.02% for the cultivated plot (Table 5). The nitrogen content, although low, differs significantly between the two plots (*p*=0.028). The average C/N ratio varies between 13.86 ± 11.66 and 25.69 ± 17.32 in the studied soils. A higher value was observed under the forest reserve. The average available phosphorous content is practically low in the two plots. Its average levels are 7.93 ± 2.09 mg/kg in the forest reserve and 9.93 ± 7.01 mg/kg in the cultivated plots (Table 4). The Mg/K ratio varies significantly between the two plots (*p*=0.002). The average values are, respectively, 5.85 ± 2.02 in the cultivated soils and 2.28 ± 1.99 in the forest reserve. For the Ca/Mg ratio, the quotients are low in both plots, with 1.89 ± 0.62 in the cultivated plot and 2.32 ± 0.59 under the forest reserve. No significant difference was observed between the two values (Table 4).

### 3.2. Degradation Rate/Vulnerability Potential

#### 3.2.1. Forest Reserve

The SDR/VP (degradation rate/vulnerability potential) weighted value for pH_H2O_, Na, ISS, and IB is 1/5 and 4/2 for BD (Table 6). This value reflects no soil degradation or vulnerability. The SDR/VP weighted at 2/4 for V reflects a slight degradation or a low vulnerability. Texture and OC, with a SDR/VP of 3/3, indicate moderate degradation or vulnerability of the soils under the reserve. Severe degradation or high vulnerability is explained by the weighted value 4/2 assigned to the SAR and N (Table 6). The SDR/VP weighted value of 4/1 for BD reflects severe degradation or high vulnerability of the soils under the forest reserve. Overall, the sum of the weighting factors of the different parameters resulted in a cumulative rating index of 27 based on eleven soil properties.

#### 3.2.2. Cultivated Plots

The various parameters that reflect the state of degradation/vulnerability potential of soils under cultivation show that five soil quality parameters indicate severe degradation or high vulnerability potential of soils (SDR/VP = 4/2). These parameters are texture, OC, ISS, N, and IB. These soil parameters (except for N), in the forest reserve, on the contrary, indicate no degradation/vulnerability (ISS, IB) and slight degradation/vulnerability (texture, OC). P, with a weighted value of SDR/VP = 3/3, reflects moderate degradation or vulnerability of cultivated soils. V, Na, and SAR have SDR/VP = 2/4, testifying a slight degradation or a low potential vulnerability of cultivated plots due to these parameters. Unlike the pH_H2O_ with SDR/VP = 1/5, which indicates that the soils run no risk of degradation/vulnerability, BD has a SDR/VP = 5/1, reflecting an extreme degradation or very high vulnerable potential of soils under cultivation, as already noted in soils under the forest reserve. The cumulative rating index estimated by the sum of the various parametric values describing the state of degradation or vulnerability of the soils under cultivation for the weighted parameters is high (35) compared to the value obtained under the forest reserve (28) (Table 7).

### 3.3. Soil Fertility

#### 3.3.1. Relationship between Soil Physicochemical Properties

In forest reserve soils, OM was significantly and positively correlated with silt (*r* = 0.640, *p* < 0.05) (Table 8). pH_H2O_ was significantly and negatively correlated with SBE (*r* = −0.921, *p* < 0.05) in the forest reserve but significantly and positively correlated with SBE (*r* = 0.815, *p* < 0.05) and K (*r* = 0.746, *p* < 0.05) in cultivated soils. In addition, there were significant and negative correlations between pH_H2O_ and N (*r* = −0.740, *p* < 0.05), pH_H2O_ and Na (*r* = −0.730, *p* < 0.05), C/N and V (*r* = −0.830, *p* < 0.05), and V with ISS (*r* = −0.733, *p* < 0.05) in the forest reserve. On the other hand, the correlation between N and SBE (*r* = 0.742, *p* < 0.05), C/N et SBE are significant and positive, also under the forest reserve. As for the physical properties, BD is significantly and negatively correlated with C/N (*r* = −0.685, *p* < 0.05). As well as sand and silt (*r* = −0.902, *p* < 0.05), silt with pHKCl (*r* = −0.716, *p* < 0.05), and clay and pH_H2O_ (*r* = −0.828, *p* < 0.05) are significantly and negatively correlated (Table 8).

In the cultivated plot, significant positive correlations were noted between pH_H2O_ and CEC (*r* = 0.805, *p* < 0.05), pHKCl and Ca (*r* = 0.749, *p* < 0.05), pH_KCl_ and K (*r* = 0.749, *p* < 0.05), pHKCl and CEC (*r* = 0.865, *p* < 0.05), pH_KCl_ and SBE (*r* = 0.817, *p* < 0.05), N and CEC (*r* = 0723, *p* < 0.05), and N and P (*r* = 0.768, *p* < 0.05) (Table 9). It was also noted between Ca and K (*r* = 0.675, *p* < 0.05), Ca and Na (*r* = 0.659, *p* < 0.05), Ca and CEC (*r* = 0.709, *p* < 0.05), Mg and V (*r* = 0.709, *p* < 0.05), and between CEC and SBE (*r* = 0.693, *p* < 0.05). In these cultivated plots, only significant and negative correlations are noted, between sand with clay (*r* = −0.814, *p* < 0.05), Ca/Mg and BD (*r* = −0.673, *p* < 0.05), and Ca/Mg and Mg/K (*r* = −0.806, *p* < 0.05) (Table 9).

#### 3.3.2. Distribution of Variables and Individuals on the Main Axes

**Figure 4:**
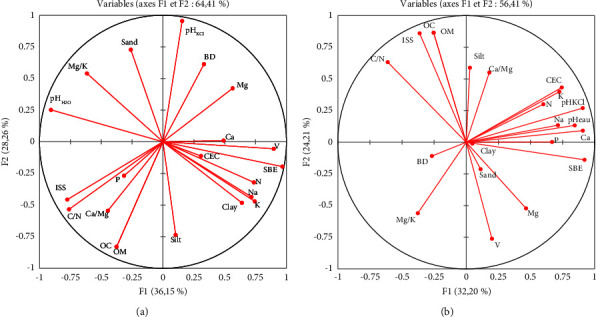
Principal component analysis of soil properties: ((a) circle of correlation under reserve; (b) circle of correlation of cultivated plot). SBE: sum of exchangeable bases; ISS: soil aggregate stability index; BD: bulk density.

The principal component analysis (PCA) of different physicochemical properties of soils shows variability in the dataset along the F1 and F2 axes. This analysis was carried out separately for the plots under the forest reserve (Figure 4(a)) and plots under cultivation (Figure 4(b)).

In the forest reserve, the discriminatory factorial analysis explains 64.41% of the variability of the dataset, with 36.15% on the F1 axis and 28.26% on F2. The F1 axis is strongly and positively represented by Ca, V, SBE, and CEC. It is weakly and positively represented by Na, N, K, and clay and likewise negatively represented by pH_H2O_, ISS, C/N, and P (Figure 4(a)). The F2 axis, meanwhile, is strongly and positively represented by sand, BD, and pH_KCl_. It is strongly negatively correlated with silt but weakly and negatively represented by OM (Figure 4(a)).

Compared to the forest reserve, the variability of the dataset on the main plan is expressed as 56.41%, with 32.20% on the F1 axis and 24.21% on F2 in the cultivated soils. Clay, P, Na, Ca, SBE, and pH_H2O_ represent strongly and positively F1, while BD represents it strongly and negatively (Figure 4(b)). Likewise, K, CEC, nitrogen, and pH_KCl_ represent weakly and positively F1. The F2 axis is strongly and positively represented by OM and silt. It is weakly represented by ISS, C/N, and Ca/Mg. It is strongly and negatively represented by V and sand but more weakly by Mg and Mg/K (Figure 4(b)).

#### 3.3.3. Fertility Level of the Studied Luvisols

The statistical analysis of the different physicochemical properties and fertility parameters as well as the balances between these different parameters permits to determine the current level of fertility of the luvisols in the locality of Sir. The studied soils had no limitations in K, V, SBE, CEC, and IF (Table 10). Available phosphorus (P) shows a moderate limitation in both cases. There is no limitation in OM in forest reserve soils, while it is moderate in cultivated soils. The limitation in nitrogen (N) is moderate in the soils under cultivation, while it is weak in the forest reserve. There is a severe limitation in ISS and IB in the cultivated soils but not in the forest reserve. Soils under the forest reserve have a stable structure contrary to cultivated soils which have a degraded structure. Table 10 summarizes two main soil classes corresponding to the level of fertility in the studied area:

- Class II groups together soils with a good level of fertility. This class characterized the fertility of soils under forest reserves. These soils have a moderate phosphorous limitation;
- Class IV includes soils with a poor level of fertility (low fertility). This class corresponds to that of soils under cultivation. They present severe limitations in ISS and IB, moderate limitations in OM and P, and weak limitations in N (Table 10).

## 4. Discussion

### 4.1. Physicochemical Characteristics of Soils

#### 4.1.1. pH, Bulk Density, Texture, and Soil Aggregate Stability Index

The pH in the two plots varies from acidic to neutral (5.2 to 7.1). These pH values are common under the Sudano-Sahelian climate [7, 23]. Sand content was very high in both plots. The highest proportions of clay and the lowest content of silt were noted in the cultivated plot. The soil aggregate stability index of the luvisols in the study area evolved from a stable structure (SI > 9%) in the forest reserve to a degraded structure in the cultivated plot (SI < 5%). These results obtained in the forest reserve are contrary to those of Martinez-Trinidad et al. [36] and Sung [37] who show that most soils in the dry tropical zone have a low soil aggregate stability index. In addition to being strongly and positively correlated with OM as many authors have observed in other parts of the world [38], the soil aggregate stability index is also positively correlated with the C/N ratio. Carbon is therefore an aggregate stabilizer [38]. Similarly, Razafimbelo [39] notes that the soil aggregate stability index increases with OM content. The low value of the soil aggregate stability index in cultivated soils can be explained, on the one hand, by the low OM content in these soils and, on the other hand, by the increase in the base saturation. The total content of exchangeable bases (SBE) in the cultivated plot was greater than that in the control soil. Its evolution is dependent on the Ca and Mg contents. The increase in the base saturation and the total content of exchangeable bases implies a decrease in the soil aggregate stability index [38]. This is confirmed by the negative correlations between the soil aggregate stability index and the total content of exchangeable bases on the one hand and with the base saturation on the other in soils under the forest reserve. The bulk density of cultivated soils is higher than that of soils under the forest reserve (2.2 against 1.59 g/cm^3^), in line with the observations already made by Getachew et al. [40]. Conversion of the forest reserve to agricultural land increases soil bulk densities, likely due to increased soil compaction. Higher bulk density means that less water is held in the soil, while lower bulk density means that soils are less compacted and can hold more water [7].

#### 4.1.2. Organic Carbon, Nitrogen, and Phosphorus

The OM content is generally low for all the studied soils. This result is consistent with the work of Koull and Halilat [41] which shows that in the Sudano-Sahelian region, OM exists but in very low quantity. Its content in cultivated soils has dropped considerably (1%). Similar results were obtained from Tsozué et al. [23] in Zamai soils, at the base of the Mandara Mountains. The decrease in OM content is due on the one hand to the continuous exploitation of the plots and on the other hand to the climatic conditions of the study area such as the high temperature and the humidity, which can lead to a rapid mineralization of the OM [42, 43]. Nitrogen contents in cultivated soils, although very low [44], decreased by 38.27%, with an average value of 0.081% in the forest reserve against 0.05% in cultivated soils. Most studies on the effects of cultivation and/or change in land use on the physicochemical properties of soils in the Sudano-Sahelian zone have come to the conclusion that tillage contributes to the loss of nutrients and reduces soil carbon sequestration [45]. The shift from natural vegetation to agricultural land has led to the loss of nitrogen [45]. The average C/N values of 13.86 and 24.30 were obtained in soils under cultivation and forest reserve. These results are consistent with those obtained from Tsozué et al. [23] under similar climate conditions. It can be seen that the C/N ratio increases considerably with the OM content, hence a significantly positive correlation between these two soil parameters. C/N ratios >12 under forest reserve (control) are an indicator of poorly decomposed OM which could be due either to the quality of OM or to the low activity of soil microorganisms [46].

#### 4.1.3. The Absorbent Complex

The average Ca and Mg contents are slightly higher in the cultivated plots, respectively 11.26 and 6.4 cmol·kg^−1^ against 10.44 and 4.93 cmol·kg^−1^ under forest reserve. These levels of Ca and Mg might be due to the pedogenetic processes involved or to the original material of this soil itself. K content evolves from very high under reserve (4.59 cmol·kg^−1^) to a low content in the cultivated plot (1.15 cmol·kg^−1^) according to the interpretation interval of Tabi et al. [44]. Similarly, Na content follows the same evolution as potassium, hence a positive and significant correlation between these two elements. The high content of these elements in the study area can be explained by the presence of parent rocks very rich in this element and the mineral inputs by rain and wind which are also very variable and the estimation of which is imprecise [47]. On the other hand, the decrease in their content in the cultivated plots would be linked to the poor texture which is dominated by the sandy fraction and therefore exposes the soil to relatively high leaching [48]. The CEC is generally high throughout the study site. Nevertheless, its value under forest reserve is higher than that of cultivated soils, with 39.25 cmol·kg^−1^ against 31.46 cmol·kg^−1^ respectively. High CEC values were on the contrary obtained in cultivated soils compared to the controls in Zouana and Zamai, also under Sudano-Sahelian climate [23, 49]. In general, the evolution of the CEC depends on the OM content and the particle size distribution. The high value of the CEC in the study area would be linked to the high activities of 2/1 type clays, in particular those of montmorillonites and sepiolites. The phosphorous content is low in all the studied plots. A decrease of 1.65 g/kg was observed in the forest reserve soils compared with the cultivated soils. When the clay content increases, phosphorous retention also increases. The low phosphorous content in the study area can be explained by the low clay content, hence the positive correlation with clay on both plots. The slight increase in the content of this element in cultivated soils could be explained by the addition of fertilizers or application of organic manure.

### 4.2. Degradation Rate/Vulnerability Potential of Soils

#### 4.2.1. Physical Soil Degradation

Based on the selected physicochemical properties, soils of cultivated plots are subjected to severe degradation or vulnerability (SDR/Vp = 4/2), while those under the forest reserve, on contrary, are moderately degraded (SDR/Vp = 3/3) due to soil texture. The severe degradation and high vulnerability of soils related to the texture in cultivated soils were also noted by Ufot Akpan [12] in soils in the coastal plains of Nigeria, characterized by a sandy loam texture. In the same direction, Tellen and Yerima [7], by evaluating the effects of tillage, fallowing, and burning on the physicochemical properties of soils in the north-west region of Cameroon, conclude that tillage leads to significant destruction of soil macroaggregates and therefore degradation of the soil structure marked by compaction. The soil aggregate stability and sealing indexes indicate no degradation of soils under the forest reserve (SDR/Vp = 1/5) and extreme degradation in cultivated soils (SDR/Vp = 5/1). The extreme degradation in cultivated soils would be linked to the erosion process which limits the development of the soil structure [50] and to the low OM content. However, these soils might have undergone the process of compaction, mainly due to erosion and human activities. The bulk density reflects severe degradation (SDR/Vp = 4/2) and very high vulnerability of luvisols both in forest reserves and extreme degradation (SDR/Vp = 5/1) and very high vulnerability in cultivated soils. The SAR values obtained in this study are, respectively, 17.77% in the forest reserve soils and 10.52% in the cultivated soils. These values between 10 and 26 are indicative of moderate to severe limitations, thus reflecting a high risk of soil degradation [51].

#### 4.2.2. Soil Chemical Degradation

The weighted value for OC showed severe degradation/vulnerability (SDR/VP = 4/2) in the cultivated plot and no degradation/vulnerability potential in the forest reserve soils (SDR/VP = 1/5). Cultivation would have led to a decrease in the content of OM in the studied soils. Similar results were obtained from Ufot Akpan [12] in Nigeria. There was a severe degradation or vulnerability (SDR/VP = 4/2) due to the nitrogen content. This is not only linked to the low OM content but also to the leaching processes and low mineralization which might have led to poor incorporation of nitrogen during the humification process [52]. Moderate degradation/vulnerability (SDR/Vp = 3/3) due to the base saturation rate and phosphorus was also noted. With regard to phosphorus, Ufot Akpan [12] obtained an extreme degradation or a very high vulnerability potential (SDR/VP = 5/1) in the coastal plains of the humid tropical rainforest in the south-east region of Nigeria. Tsozué and Azinwi [4] obtained severe degradation or high vulnerability due to this element in the humid tropical mountainous zone of the western zone of Cameroon. The low content of phosphorus in the studied soils would be linked to the erosion process and the poor management of crop residues or even the low retention capacity of soil in this element. The best quality indicator of the luvisols in the locality of Sir is pH, while bulk density is the parameter reflecting the poor quality of these soils. The cumulative rating index of the soils under the forest reserve is 27/27 against 35/35 for the soils under cultivation, reflecting a more accentuated physical and chemical degradation in the cultivated soils.

### 4.3. Soil Fertility Levels

#### 4.3.1. The Absorbent Complex and Soil pH

pH is a key parameter in the chemical composition of the soil which determines the availability of nutrients for plants and soil microorganisms [53]. The pH of the studied soils varies between 5.20 and 7.10. This corresponds, respectively, to slightly acidic and neutral pH. This indicates that the chemical (bioavailability of nutrients) and microbiological reactions in these soils are proceeding properly [54]. Similar results were obtained in the Damara region of the Central African Republic [55]. Even more, Delaunois et al. [56] also showed that a pH of 5.5 to 7.5 is a minimum for the development of all crops. The average values of CEC obtained both in the forest reserve and in cultivated plots are high (greater than 25 cmol·kg^−1^). This does not constitute a limitation, leading to the conclusion that the level of fertility is good with regard to the CEC in all the studied areas, in line with the observations already made by Nguemezi et al. [5] in the dystric vitric andosols at Tombel area in the south-west part of Cameroon. The average Ca contents of 10.44 cmol·kg^−1^ in the forest reserve and 11.25 cmol·kg^−1^ in the cultivated plots are considered to be quite good in all the plots. Indeed, the majority of plants develop normally with a critical threshold of 5 cmol·kg^−1^ of soil, thus excluding the hypothesis of a low Ca content in the studied soil [57]. K, which averages of 12.67 cmol·kg^−1^ in the forest reserve and 1.15 cmol·kg^−1^ in cultivated plots, is a vital element in the formation of chlorophyll and the translocation of starch in plants and is essential for the formation of lipids [58]. Magnesium content values are high both in forest reserve soils (4.93 cmol·kg^−1^) and in cultivated soils (6.4 cmol·kg^−1^). The absence of limitations indicates a level of fertility favorable to the development of plants in the study area.

#### 4.3.2. Organic Matter and Phosphorus

OM allows the long-term maintenance of soil fertility and therefore sustainable agricultural production, due to its physical, chemical, and biological effects [59, 60]. Its mineralization is a potential source of N and P to plants. In the study area, the OM content is low (1%) in soils under cultivation against 2.86% in the soils under the forest reserve. Its average content close to 3% in the forest reserve soils testifies a very good level of fertility, similar to results obtained by Nguemezi et al. [5] with OM content greater than 2% in the different soil groups. The low OM content (1%) in the cultivated plots in the study area would reflect a poor state of soil fertility [13]. The phosphorous contents of 8.272 g/kg under the forest reserve and 9.93 g/kg in the cultivated plots are severely limited and reflect deficiencies as fertilizing elements. These deficiencies in available phosphorus were already noted in the evolution of soil fertility in the Sudano-Sahelian zone of northern Cameroon [61]. Nevertheless, in many cases, phosphorus can be abundant in the soil, but deficiencies can be observed in crops due to regression forms. These soils require thus fertilizer inputs and a short-term correction to support good agricultural development.

#### 4.3.3. Soil Aggregate Stability Index, Forest Index, and Sealing Index

Aggregate distributions and soil aggregate stability index are indicators of soil fertility [62]. Soil aggregate stability mainly reduces the risks of soil erosion and compaction [50]. The difference in the soil aggregate stability index observed in the two plots is related to the OM rate and the C/N ratio, confirmed by the significant and positive correlations noted between these soil parameters. The Forest index (FI) is a parameter which helps evaluate the nutrient content in the soil. Its value is 2.17 under the forest reserve against 2.79 in cultivated soils, indicating good nutrient reserves in the studied soils [1, 5]. The sealing index value in the forest reserve is −0.608 against 305.07 in the cultivated soils. This parameter determines the degree of soil sealing linked to the risk of erosion and the process of compaction [33]. However, it measures the resistance of soils to root growth and stem expansion [1]. It appears that the luvisols under the forest reserve are not subjected to the risk of erosion or compaction. The very high value of the sealing index obtained in the cultivated plots testifies their waterproofing which might be linked to the processes of erosion and compaction as the result of ploughing and/or climatic conditions. In order to enhance the current agricultural potential of soil under cultivation, organic matter content should be restored through the management of crop residue spreading, compost, green manure, and farmyard manure. Nutrient levels could be increased by combining mineral and organic fertilization. The low phosphorous content should be improved by the application of phosphorous fertilizers.

## 5. Conclusion

The present study was designed to analyse the current character of luvisols in the locality of Sir in the Mandara Mountains and evaluate their degradation rate/vulnerability potential and fertility status. The analysis was based on the comparison of the physicochemical characteristics of soils in two contrasted plots, one under forest reserve and the other under cultivation, with different management systems. Soils under the forest reserve are subjected to severe degradation or high vulnerability potential due to bulk density, nitrogen, and sodium absorption ratio. Soils under cultivation are characterized by severe degradation or high vulnerability potential due to texture, OM, soil aggregate stability index, sealing index, nitrogen, and bulk density. The best indicator of the good quality of the luvisols of Sir is the pH. Soils under the forest reserve have a very good level of fertility, while the soils under cultivation are characterized by a low level of fertility. The results of this study will serve in the development of strategies for the management and restoration of agricultural land in the Sudano-Sahelian zone.

## Figures and Tables

**Table 1:** Historical characteristics of plots.

	Samples	Coordinates	Surface state	Land management practices
Forest reserve	*E*_1_	10°35′50″N/13°37′0.064″E	Virgin	Grazing
	*E*_2_	10°35′46.8″N/13°37′0.074″E		
	*E*_3_	10°35′41.6″N/13°37′08.9″E		
	*E*_4_	10°35′38.5″N/13°37′07.3″E		
	*E*_5_	10°35′33.5″N/13°37′02.1″E		
	*E*_6_	10°35′40.9″N/13°37′00.3″E		
	*E*_7_	10°35′48.3″N/13°37′00.9″E		
	*E*_8_	10°35′44.8″N/13°37′02.3″E		
	*E*_9_	10°35′48.5″N/13°37′03.4″E		
	*E*_10_	10°35′51.5″N/13°37′04.8″E		

Cultivated plot	*E*_11_	10°35′54.2″N/13°37′04.8″E	Cultivation of maize and soybean	(i) Application of organic manure (ii) Fallow (iii) Mulching (iv) Crop rotation (v) Crop association (vi) Weeding (vii) Ploughing (viii) Ridging (ix) Burning (x) Direct seeding (xi) Application of chemical inputs
	*E*_12_	10°35′57.2″N/13°37′04.9″E	Cultivation of maize	
	*E*_13_	10°36′02.2″N/13°37′07.6″E	Cultivation of rice	
	*E*_14_	10°36′10.6″N/13°37′11.1″E	Cultivation of millet and groundnut	
	*E*_15_	10°36′05.8″N/13°37′21.95″E	Cultivation of maize	
	*E*_16_	10°36′02.1″N/13°37′32.4″E	Cultivation of cowpea	
	*E*_17_	10°36′00.9″N/13°37′34.3″E	Cultivation of sorrel	
	*E*_18_	10°35′36.7″N/13°37′32.6″E	Cultivation of millet	
	*E*_19_	10°35′55.7″N/13°37′32.6″E	Cultivation of soybean	
	*E*_20_	10°35′55.5 N/13°37′20.4″E	Cultivation of potato	

**Table 2:** Physicochemical characteristics and summary statistics of the soil profile.

Depth (cm)	0–25	25–50	50–75	75–100	100–125	Min	Max	Mean	SD	CV
Sand	74.70	69.70	59.70	72.70	79.70	59.70	79.70	71.30	7.44	0.1
Silt	17.40	16.40	21.40	15.40	12.40	12.40	21.40	16.60	3.27	0.2
Clay	7.90	13.90	18.90	11.90	7.90	7.90	18.90	12.10	4.60	0.38
pH_H2O_	6.60	6.00	6.50	6.70	7.20	6.00	7.20	6.60	0.43	0.07
pH_KCl_	4.70	4.00	4.10	4.20	4.20	4.00	4.70	4.24	0.27	0.06
OC (%)	1.90	0.17	0.52	1.11	1.60	0.17	1.90	1.06	0.72	0.68
OM (%)	3.27	0.30	0.89	1.91	2.76	0.30	3.27	1.83	1.24	0.68
N (%)	0.04	0.01	0.03	0.03	0.02	0.01	0.04	0.03	0.01	0.41
C/N	45.13	14.48	18.46	44.31	65.31	14.48	65.31	37.54	21.04	0.56
Ca (cmol·kg^−1^)	7.68	8.48	9.28	10.96	7.60	7.60	10.96	8.80	1.39	0.16
Mg (cmol·kg^−1^)	2.48	4.88	5.12	4.72	6.32	2.48	6.32	4.70	1.39	0.3
K (cmol·kg^−1^)	6.04	1.56	0.91	1.01	0.41	0.41	6.04	1.98	2.30	1.16
Na (cmol·kg^−1^)	1.27	0.97	0.77	0.87	0.57	0.57	1.27	0.89	0.26	0.29
SBE (cmol·kg^−1^)	17.47	15.89	16.08	17.56	14.90	14.90	17.56	16.38	1.13	0.07
CEC (cmol.kg^−1^)	38.24	42.88	52.48	24.80	20.32	20.32	52.48	35.74	13.18	0.37
P (mg/kg)	12.50	8.71	4.92	3.92	4.42	3.92	12.50	6.89	3.66	0.53
V (%)	45.67	37.04	30.64	70.79	73.31	30.64	73.31	51.49	19.53	0.38
ISS (%)	12.92	0.98	2.21	7.00	13.59	0.98	13.59	7.34	5.85	0.8
Gravel (%)	15.00	7.00	25.00	25.00	25.00	7.00	25.00	19.40	8.17	0.42

Min: minimum; Max: maximum; SD: standard deviation; CV: coefficient of variation; SBE: sum of exchangeable bases; ISS: soil aggregate stability index.

**Table 3:** Physicochemical characteristics and summary statistics of the soils under the forest reserve.

Samples	E1	E2	E3	E4	E5	E6	E7	E8	E9	E10	Min	Max	Mean	SD	CV
Sand	67.6	70.6	80.6	60.6	78.6	73.6	78	74.7	75.7	73.7	60.6	80.6	73.88	5.84	0.08
Silt	22	22	12	26	13	17	13.6	17.4	18.4	18.4	12	26	17.67	4.34	0.25
Clay	10.4	7.4	7.4	13.4	8.4	9.4	8.4	7.9	5.9	7.9	5.9	13.4	8.45	2.07	0.24
pH_H2O_	6.1	6.5	6.5	5.2	6.2	6.4	6.4	6.6	6.8	7.1	5.2	7.1	6.39	0.48	0.07
pH_KCl_	4.9	5.3	5.4	3.9	5.3	5.3	5.5	4.7	5	5.2	3.9	5.5	5.05	0.45	0.09
OC (%)	2.23	1.21	1.34	2.28	1.12	1.45	0.34	1.9	2.09	2.68	0.34	2.68	1.6	0.69	0.43
OM (%)	3.85	2.09	2.31	3.93	1.92	2.51	0.59	3.27	3.61	4.63	0.59	4.63	2.76	1.2	0.43
N (%)	0.1	0.1	0.07	0.13	0.1	0.07	0.08	0.04	0.09	0.04	0.02	0.13	0.08	0.03	0.42
C/N	22.44	12.46	20.14	17.04	11.54	21.65	4.22	45.13	24.5	63.88	4.22	63.9	25.69	17.32	0.67
Ca (cmol·kg^−1^)	13.52	8.8	7.92	9.76	10.96	12.16	13.5	7.68	9.52	10.56	7.68	13.52	10.41	2.01	0.19
Mg (cmol·kg^−1^)	6.4	6.32	5.6	3.76	4.72	4.96	6.56	2.48	3.6	4.88	2.48	6.56	4.76	1.39	0.29
K (cmol·kg-1)	4.32	6	5.77	10.94	8.29	1.3	1.66	6.04	0.73	0.91	0.73	10.94	4.59	3.45	0.71
Na (cmol·kg^−1^)	3.07	1.17	1.27	1.67	1.57	0.87	0.97	1.27	0.77	0.87	0.77	3.07	1.32	0.65	0.49
SBE (cmol·kg^−1^)	27.31	22.29	20.56	26.13	25.54	19.29	22.7	17.47	14.62	17.22	14.62	27.31	21.31	4.22	0.18
CEC (cmol·kg^−1^)	71.44	42.72	39.52	33.28	39.52	36.16	25.4	38.24	28.64	37.6	25.44	71.44	38.15	12.39	0.32
P (mg/kg)	8.52	6.78	5.97	7.15	7.71	9.95	7.9	12.5	8.89	7.34	4.54	12.5	7.93	2.09	0.26
Ca/Mg	2.11	1.39	1.41	2.6	2.32	2.45	2.06	3.1	2.64	2.16	1.39	3.26	2.32	0.59	0.25
Mg/K	1.48	1.05	0.97	0.34	0.57	3.81	3.94	0.41	4.95	5.36	0.34	5.36	2.28	1.99	0.82
V (%)	38.22	52.19	52.03	78.51	64.63	53.35	89.3	45.67	51.04	45.8	38.22	89.3	57.07	15.87	0.26
ISS (%)	11.87	7.11	11.91	9.97	8.98	9.5	2.66	12.92	14.84	17.59	2.66	17.6	10.48	4.02	0.38
IB (%)	−2.8	−6.27	−4.89	−2.9	−7.09	−5.03	31.1	−3.23	−2.76	−2.18	−7.09	31.1	−0.61	11.25	−17.6
IF	1.41	1.7	3.35	0.93	2.89	2.05	2.77	2.21	2.36	2.07	0.93	3.35	2.17	0.72	0.31
BD (g/cm^3^)	0.79	1.45	1.94	1.74	2.11	1.88	1.68	1.31	1.56	1.5	0.79	2.11	1.59	0.37	0.22

Min: minimum; Max: maximum; SD: standard deviation; CV: coefficient of variation; SBE: sum of exchangeable bases; IF: Forestier index; ISS: soil aggregate stability index; IB: soil sealing index.

**Table 4:** Physicochemical characteristics and summary statistics of the soils in the cultivated plot.

Samples	E11	E12	E13	E14	E15	E16	E17	E18	E19	E20	Min	Max	Mean	ET	CV
Sand	79	76	75	80	78	76	86	78	68	76	68	86	77.2	4.52	0.06
Silt	14.6	14.6	13	11.6	14.6	16.6	7.6	12.6	18.6	12.6	7.6	18.6	13.6	2.98	0.22
Clay	6.4	9.4	12	8.4	7.4	7.4	6.4	9.4	13.4	11.4	6.4	13.4	9.2	2.49	0.27
pH_H2O_	6.5	5.7	6	6.6	7.1	6.3	6.2	6.7	6.7	5.2	5.2	7.1	6.3	0.55	0.09
pH_KCl_	5	4.8	4.8	5.4	6.1	5.1	4.5	5.6	5.4	4.1	4.1	6.1	5.08	0.58	0.11
OC (%)	0.97	0.73	0.1	0.24	0.28	1.02	0.1	0.82	0.78	1.21	0.1	1.21	0.63	0.41	0.65
OM (%)	1.67	1.25	0.2	0.42	0.48	1.76	0.17	1.42	1.34	2.09	0.17	2.09	1.08	0.7	0.65
N (%)	0.02	0.03	0.1	0.05	0.09	0.06	0.02	0.08	0.05	0.06	0.02	0.09	0.05	0.02	0.44
C/N	39.6	23.1	1.9	4.56	3.23	15.8	5.54	9.73	14.8	20.4	1.88	39.58	13.86	11.66	0.84
Ca (cmol·kg^−1^)	10.2	9.6	12	12.8	12.2	10.7	7.76	16.6	12.2	8.32	7.76	16.56	11.26	2.55	0.23
Mg (cmol·kg^−1^)	3.12	4.48	5.9	6.96	6.56	6.8	8.56	7.6	9.36	4.64	3.12	9.36	6.4	1.92	0.3
K (cmol·kg^−1^)	1.54	0.88	1	1.42	1.19	1.19	0.88	1.54	1.19	0.7	0.7	1.54	1.15	0.29	0.25
Na (cmol·kg^−1^)	0.97	0.77	0.9	1.07	0.87	0.97	0.87	1.07	0.87	0.77	0.77	1.07	0.91	0.11	0.12
SBE (cmol·kg^−1^)	15.8	15.7	20	22.3	20.8	19.7	18.07	26.8	23.6	14.4	14.43	26.77	19.72	3.86	0.2
CEC (cmol·kg^−1^)	27	25.9	26	27.2	66.4	27.2	21.28	35.8	30.3	27.2	21.28	66.4	31.46	12.8	0.41
P (mg/kg)	4.54	6.84	8	13.7	28.5	10.3	6.1	7.65	6.78	6.78	4.54	28.53	9.93	7.01	0.71
Ca/Mg	3.26	2.14	2.1	1.84	1.85	1.58	0.91	2.18	1.3	1.79	0.91	3.26	1.89	0.62	0.33
Mg/K	2.03	5.08	6	4.91	5.51	5.71	9.71	4.94	7.86	6.65	2.03	9.71	5.85	2.02	0.35
V (%)	58.4	60.7	76	81.8	31.3	72.4	84.92	74.9	78	53.1	31.29	84.92	67.14	16.38	0.24
ISS (%)	7.96	5.22	0.8	2.09	2.19	7.31	1.19	6.46	4.18	8.71	0.84	8.71	4.62	2.93	0.64
IB (%)	−7.8	−25	7.5	18.4	31.1	−7.9	16.21	−16	3042	−7.6	−24.9	3041.8	305.08	961.7	3.15
IF	2.97	2.41	2.3	3.2	2.77	2.41	5.28	2.77	1.45	2.41	1.45	5.28	2.79	1	0.36
BD (g/cm^3^)	1.85	1.61	1.5	1.68	1.8	2.6	3.44	1.88	3.24	2.7	1.49	3.44	2.23	0.71	0.32

Min: minimum; Max: maximum; SD: standard deviation; CV: coefficient of variation; SBE: sum of exchangeable bases; IF: Forestier index; ISS: soil aggregate stability index; IB: soil sealing index.

**Table 5:** Mean soil characteristics variability of in the study area.

Soil characteristics	Forest reserve	Cultivated plot	*p* value
Sand (%)	73.88 ± 5.84a	77.2 ± 4.52a	0.095
Silt (%)	17.67 ± 4.34a	13.6 ± 2.98b	0.034^*∗*^
Clay (%)	8.45 ± 2.07a	9.2 ± 2.49a	0.732
pH_H2O_	6.38 ± 0.48a	6.3 ± 0.55a	0.82
pH_KCl_	5.05 ± 0.45a	5.08 ± 0.58a	0.97
OC (%)	1.66 ± 0.69a	0.63 ± 0.41b	0.001^*∗*^
OM (%)	2.76 ± 1.20a	1.08 ± 0.70b	0.001^*∗*^
N (%)	0.081 ± 0.03a	0.05 ± 0.02b	0.028^*∗*^
C/N	25.69 ± 17.32a	13.86 ± 11.66a	0.112
Ca (cmol·kg^−1^)	10.44 ± 2.01a	11.26 ± 2.55a	0.495
Mg (cmol·kg^−1^)	4.76 ± 1.39a	6.4 ± 1.92a	0.076
K (cmol·kg^−1^)	4.59 ± 3.45a	1.15 ± 0.29b	0.019^*∗*^
Na (cmol·kg^−1^)	1.32 ± 0.65a	0.91 ± 0.11b	0.046^*∗*^
CEC (cmol·kg^−1^)	38.15 ± 12.39a	31.46 ± 12.80b	0.023^*∗*^
SBE (cmol·kg^−1^)	21.31 ± 24.22a	19.716 ± 3.86a	0.406
P (mg/kg)	7.93 ± 2.09a	9.93 ± 7.01a	0.705
BD (g/cm^3^)	1.59 ± 0.37a	2.22 ± 0.71a	0.256
Ca/Mg	2.32 ± 0.59a	1.893 ± 0.62a	0.131
Mg/K	2.28 ± 1.99a	5.845 ± 2.02a	0.002^*∗*^
ISS (%)	10.48 ± 4.02a	4.615 ± 2.93b	0.002^*∗*^
V (%)	57.07 ± 15.87a	67.144 ± 16.38a	0.406

^*∗*^Significant at *p* < 0.05. Numbers followed by different lowercase letters within the same line have significant differences (*p* < 0.05); SBE: sum of exchangeable bases; ISS: soil aggregate stability index; BD: bulk density.

**Table 6:** Rating scheme for soil degradation rates (SDR) and vulnerability potential (Vp) of selected soil quality indicators under the forest reserve.

Properties	Mean	SDR	VP	SDR/Vp
Texture	Sandy loam	3	3	3/3
pH_H2O_	6.38 ± 0.503	1	5	1/5
P (mg/kg)	8.272 ± 1.86	3	3	3/3
Na (cmol·kg^−1^)	8.483 ± 12.67	1	5	1/5
OC (%)	1.66 ± 0.698	3	3	3/3
BD (g/cm^3^)	1.59 ± 0.37	4	2	4/2
ISS (%)	10.73 ± 4.148	1	5	1/5
IB (%)	−0.608 ± 11.25	1	5	1/5
N (%)	0.081 ± 0.028	4	2	4/2
V (%)	57.07 ± 15.87	2	4	2/4
SAR (%)	17.769 ± 7.34	4	2	4/2
Cumulative rating index		27	27	27/27

IF: Forestier index; ISS: soil aggregate stability index; IB: soil sealing index; BD: bulk density; SAR: sodium absorption ratio.

**Table 7:** Rating scheme for soil degradation rates (SDR) and vulnerability potential (Vp) of selected soil quality indicators in the cultivated plot.

Properties	Mean	SDR	VP	SDR/Vp
Texture	Loamy sand	4	2	4/2
pH_H2O_	6.3 ± 0.554	1	5	1/5
P (mg/kg)	9.925 ± 7.012	3	3	3/3
Na (cmol·kg^−1^)	1.15 ± 0.29	2	4	2/4
OC (%)	0.62 ± 0.407	4	2	4/2
BD (g/cm^3^)	2.22 ± 0.70	5	1	5/1
ISS (%)	4.615 ± 2.933	4	2	4/2
IB (%)	305.07 ± 961.72	4	2	4/2
N (%)	0.05 ± 0.024	4	2	4/2
V (%)	67.144 ± 16.38	2	4	2/4
SAR (%)	10.572 ± 18.34	2	4	2/4
Cumulative rating index		35	35	35/35

IF: Forestier index; ISS: soil aggregate stability index; IB: soil sealing index; BD: bulk density; SAR: sodium absorption ratio.

**Table 8:** Spearman correlation matrix for linear relationships between soil properties under the forest reserve.

Variables	Sand	Silt	Clay	pH_H2O_	pH_KCl_	OC	OM	N	C/N	Ca	Mg	K	Na	CEC	P	BD	Ca/Mg	Mg/K	ISS	SBE	V
Sand	1																				
Silt	−0.902^*∗*^	1																			
Clay	−0.526	0.252	1																		
pH_H2O_	0.354	−0.156	−0.828^*∗*^	1																	
pH_KCl_	0.632	−0.716^*∗*^	−0.291	0.099	1																
OC	−0.576	0.640^*∗*^	0.190	0.146	−0.779^*∗*^	1															
OM	−0.576	0.640^*∗*^	0.190	0.146	−0.779^*∗*^	1.000^*∗*^	1														
N	−0.523	0.566	0.436	−0.740^*∗*^	−0.258	0.006	0.006	1													
C/N	−0.188	0.250	−0.226	0.598	−0.558	0.770^*∗*^	0.770^*∗*^	−0.498	1												
Ca	−0.176	0.003	0.641^*∗*^	−0.517	0.222	−0.085	−0.085	0.317	−0.298	1											
Mg	−0.030	−0.104	0.171	−0.293	0.632	−0.406	−0.406	0.201	−0.479	0.559	1										
K	−0.139	0.085	0.361	−0.567	−0.252	−0.188	−0.188	0.469	−0.442	−0.273	−0.188	1									
Na	−0.256	0.181	0.563	−0.730^*∗*^	−0.333	0.037	0.037	0.520	−0.274	0.061	0.098	0.976^*∗*^	1								
CEC	−0.127	0.067	−0.055	−0.116	−0.031	−0.018	−0.018	0.146	0.067	−0.182	0.261	0.539	0.549	1							
P	−0.055	−0.037	0.153	0.104	−0.313	0.091	0.091	−0.249	0.406	0.207	−0.333	−0.139	−0.238	−0.297	1						
BD	0.515	−0.622	0.080	−0.262	0.485	−0.406	0.099	−0.503	0.042	−0.067	0.176	−0.049	0.079	−0.267	−0.309	1					
Ca/Mg	−0.115	0.146	0.147	0.085	−0.681^*∗*^	0.430	0.430	−0.134	0.479	−0.182	−0.867^*∗*^	−0.055	−0.104	−0.455	0.685^*∗*^	−0.430	1				
Mg/k	0.115	−0.043	−0312	0.518	0.337	0.115	0.115	−0.370	0.297	0.394	0.321	−0.952^*∗*^	−0.756^*∗*^	−0.316	0.176	−0.188	−0.188	1			
ISS	0.030	0.134	−0.343	0.604	−0.509	0.758^*∗*^	0.758^*∗*^	−0.468	0.915^*∗*^	−0.413	−0.564	−0.309	−0.220	0.006	0.176	−0.588	0.455	0.164	1		
SBE	−0.321	0.195	0.710^*∗*^	−0.921^*∗*^	−0.031	−0.188	−0.188	0.742^*∗*^	−0.624	0.474	0.467	0.842^*∗*^	0.835^*∗*^	0.333	−0.297	0.152	−0.345	−0.721^*∗*^	−0.612	1	
V	0.139	−0.226	0.287	−0.457	0.448	−0.539	−0.539	0.383	−0.830^*∗*^	0.224	0.188	0.285	0.037	−0.498	−0.297	0.685^*∗*^	−0.152	−0.176	−0.733^*∗*^	0.333	1

^*∗*^significant at *p* < 0.05; SBE: sum of exchangeable bases; ISS: soil aggregate stability index; BD: bulk density.

**Table 9:** Spearman correlation matrix for linear relationships between soil properties in the cultivated plot.

Variables	Sand	Silt	Clay	pH_H2O_	pH_KCl_	OC	OM	N	C/N	Ca	Mg	K	Na	CEC	P	BD	Ca/Mg	Mg/K	ISS	SBE	V
Sand	1																				
Silt	−0.618	1																			
Clay	−0.814^*∗*^	0.188	1																		
pH_H2O_	0.188	0.268	−0.144	1																	
pH_KCl_	0.062	0.338	−0.034	0.939^*∗*^	1																
OC	−0.295	0.503	0.092	−0.097	−0.018	1															
OM	−0.295	0.503	0.092	−0.097	−0.018	1.000^*∗*^	1														
N	−0.284	0.137	0.267	0.378	0.560	0.170	0.170	1													
C/N	−0.074	0.416	−0.135	−0.340	−0.329	0.721^*∗*^	0.721^*∗*^	−0.480	1												
Ca	−0.120	0.012	0.340	0.619	0.749^*∗*^	−0.140	−0.140	0.622	−0.529	1											
Mg	0.062	−0.118	0.135	0.492	0.366	−0.321	−0.321	0.055	−0.455	0.304	1										
K	0.292	0.177	−0.255	0.746^*∗*^	0.749^*∗*^	0.136	0.136	0.211	−0.019	0.675^*∗*^	0.142	1									
Na	0.442	−0.129	−0.331	0.589	0.597	0.013	0.013	0.209	−0.189	0.659^*∗*^	0.328	0.881^*∗*^	1								
CEC	−0.130	0.374	0.169	0.805^*∗*^	0.865^*∗*^	0.310	0.310	0.723^*∗*^	−0.237	0.640^*∗*^	0.340	0.551	0.418	1							
P	−0.074	0.031	0.092	0.363	0.584	−0.201	−0.201	0.768^*∗*^	−0.614	0.613	0.109	0.173	0.272	0.543	1						
BD	0.105	0.043	−0.141	0.073	−0.159	0.285	0.285	−0.286	0.248	−0.438	0.552	−0.148	−0.051	0.067	−0.511	1					
Ca/Mg	0.074	0.043	−0.049	0.073	0.213	0.164	0.164	0.188	0.212	0.347	−0.636	0.475	0.227	0.055	0.030	−0.673^*∗*^	1				
Mg/K	−0.363	−0.031	0.306	−0.286	−0.433	−0.188	−0.188	−0.122	−0.224	−0.432	0.455	−0.710^*∗*^	−0.568	−0.225	−0.231	0.588	−0.806^*∗*^	1			
ISS	−0.111	0.404	−0.067	−0.152	−0.098	0.964^*∗*^	0.964^*∗*^	0.012	0.830^*∗*^	−0.280	−0.418	0.117	−0.025	0.182	−0.328	0.285	0.248	−0.273	1		
SBE	−0.037	0.056	0.245	0.815^*∗*^	0.817^*∗*^	−0.261	−0.261	0.462	−0.576	0.857^*∗*^	0.709^*∗*^	0.630	0.631	0.693^*∗*^	0.456	−0.018	−0.018	−0.115	−0.394	1	
V	0.172	−0.416	0.086	0.043	−0.067	−0.588	−0.588	−0.383	−0.382	0.195	0.709^*∗*^	0.049	0.328	−0.274	−0.103	0.212	−0.455	0.297	−0.624	0.418	1

^*∗*^Significant at *p* < 0.05; SBE: sum of exchangeable bases; ISS: soil aggregate stability index; BD: bulk density.

**Table 10:** Physicochemical limitation degree and fertility level of soils.

Site	Forest reserve	Cultivated plot
OM (%)	I	III
N (%)	I	II
ISS (%)	I	IV
V (%)	I	I
K (cmol·kg^−1^)	I	I
SBE (cmol·kg^−1^)	I	I
CEC (cmol·kg^−1^)	I	I
P (mg/kg)	III	III
IF	I	I
IB (%)	I	IV
Limiting factors	P	OM, N, P, ISS, IB
Soil classes	II	IV
Fertility level	Good	Poor

SBE: sum of exchangeable bases; IF: Forestier index; ISS: soil aggregate stability index; IB: soil sealing index.

## Data Availability

All the data used to support the findings of this study are included in this paper.

## References

[1] Tedontsah V. P. L., Mbog M. B., Ngon Ngon G. F. (2022). Physicochemical properties and fertility assessment of soils in foumban (west Cameroon). *Applied and Environmental Soil Science*.

[2] Pozza L. E., Field D. J. (2020). The science of soil security and food security. *Soil Security*.

[3] Mulumba L. N., Lal R. (2008). Mulching effects on selected soil physical properties. *Soil and Tillage Research*.

[4] Tsozué D., T Azinwi P. (2016). Physicochemical characteristics, degradation rate and vulnerability potential of Mount Bambouto soils in Western Highlands of Cameroon. *Syllabus Review*.

[5] Nguemezi C., Tematio P., Yemefack M., Tsozué D., Silatsa T. B. F. (2020). Soil quality and soil fertility status in major soil groups at the Tombel area, South-West Cameroon. *Heliyon*.

[6] Gomiero T. (2016). Soil degradation, land scarcity and food security: reviewing a complex challenge. *Sustainability*.

[7] Tellen V. A., Yerima B. P. K. (2018). Effects of land use change on soil physicochemical properties in selected areas in the North West region of Cameroon. *Environmental Systems Research*.

[8] Tsozué D., Haiwe B. R., Louleo J., Nghonda J. P. (2014). Local initiatives of land rehabilitation in the sudano-sahelian region: case of harde soils in the far north region of Cameroon. *Open Journal of Soil Science*.

[9] Tsozué D., Mana P., Louléo J. (2016). Use of no-till system on straw by cotton producers in Cameroon. *British Journal of Applied Science & Technology*.

[10] Kombate A. (2013). Assessment of the quality of the soils of the Guyanese forest with a view to a change of use: cartographic study of the lands of the Pas de Nancibo.

[11] Raunet M., Naudin K. (2006). Combating desertification: the contribution of direct-seeding mulch-based cropping systems. *Thematic files, Montpellier, France*.

[12] Ufot Akpan A. (2012). Physicochemical properties, degradation rate and vulnerability potential of soils formed on coastal plain sands in southeast, Nigeria. *International Journal of Agricultural Research*.

[13] Amonmide I., Dagbenonbakin G., Agbangba C., Akponikpe P. (2019). Contribution à l’évaluation du niveau de fertilité des sols dans les systèmes de culture à base du coton au Bénin. *International Journal of Brain and Cognitive Sciences*.

[14] Kodji P., Ibrahima A., Ibrahima A. (2021). Impacts of refugees and climate change on agricultural yields in the sahelian zone of Minawao, Cameroon. *Environmental Challenges*.

[15] Njomaha C., Olina B. J. P., Abou Abba A., Oumarou B. (2010). Diffusion of direct-seeding mulch-based cropping systems in North Cameroon: adoption constraints and prospects. *Symposium proceedings, Ouagadougou, Burkina Faso*.

[16] Joko T., Anggoro S., Sunoko H. R., Rachmawati S. (2017). Pesticides usage in the soil quality degradation potential in Wanasari Subdistrict, Brebes, Indonesia. *Applied and Environmental Soil Science*.

[17] Tchouhla R., Dedzo M. G., Chako-Tchamabé B. (2023). Petrogenesis of lavas from Mokolo-Kosséhone region, northernmost segment of the Cameroon Volcanic Line: constraints from major/trace elements and Sr-Nd-Pb isotopic data. *Geosciences Journal*.

[18] Fantong W. Y., Ngappe C., Banseka H. S. (2019). Defluoridation of fluoride-rich groundwater in Mayo tsanaga river BasinCameroon using locally produced bone char. *Journal of the Cameroon Academy of Sciences*.

[19] Moksia F., Yougouda H., Jeanne Flore N. (2019). Diversity and socio-economic value of wild edible plants in the mounts Mandara region, Cameroon. *International Journal of Sciences*.

[20] Ngounouno I., Déruelle B., Demaiffe D. (2000). Petrology of the bimodal cenozoic volcanism of the Kapsiki Plateau (northernmost Cameroon, central Africa). *Journal of Volcanology and Geothermal Research*.

[21] Tamen J. C., Nkoumbou C., Reusser E., Tchoua F. (2015). Petrology and geochemistry of mantle xenoliths from the Kapsiki Plateau (Cameroon volcanic line): implications for lithospheric upwelling. *Journal of African Earth Sciences*.

[22] Gountié Dedzo M., Asaah A. N. E., Martial Fozing E. (2019). Petrology and geochemistry of lavas from Gawar, Minawao and Zamay volcanoes of the northern segment of the Cameroon Volcanic Line (Central Africa): constraints on mantle source and geochemical evolution. *Journal of African Earth Sciences*.

[23] Tsozué D., Nafissa B., Basga S. D., Balna J. (2020). Soil change in arenosols under long term cultivation in the sudano-sahelian zone of Cameroon. *Geoderma Regional*.

[24] Tsozué D., Noubissie N. M. M., Mamdem E. L. T., Basga S. D., Oyono D. L. B. (2021). Effects of environmental factors and soil properties on soil organic carbon stock in a natural dry tropical area of Cameroon. *SOIL*.

[25] Issine A., Tsozué D., Nzeukou A. N., Yongue R. F., Kagonbé B. P. (2022). Soil characteristics and pedoclimatic evaluation of rainfed sorghum (sorghum bicolor (L.) moench) in the mayo-lemié division, south-western Chad. *Applied and Environmental Soil Science*.

[26] Usda (2004). *Soil Survey Laboratory Methods Manual, Soil Survey Investigation Report No. 42*.

[27] Guitián F., Carballas T. (1976). *Soil Analysis Techniques*.

[28] Lal R. (1994). Methods and guidelines for assessing sustainability use of soil and water resources in the tropic. *Soil Management Support Technical Monograph*.

[29] Pauwels J., Van Ranst E., Verloo M., Mvondo A. (1992). *Manuel de Laboratoire de Pédologie-méthodes d’analyses de sols et de plantes; equipment et gestion des stocks de verrerie et de produits chimiques*.

[30] Walkley A., Black I. A. (1934). An examination of the Degtjarettt method of determining soil organic matter and proposed modification of the chromic acid titration method soil. *Soil Science*.

[31] Bray R. H., Kurtz L. T. (1945). *Soil Science and Conservation*.

[32] Pieri G. M. J. C. (1992). *Fertility of Soils: A Future for Farming in the West African Savannah*.

[33] Remy C. J., Marin-Laflèche A. (1974). Soil analysis: carrying out a program automatic interpretation. *Annales Agronomiques*.

[34] Forestier J. (1960). Fertility of coffee plantation soils in the CAR. *Agronomie Tropicale*.

[35] Quemada M., Cabrera M. L. (1995). CERES-N model predictions of nitrogen mineralized from cover crop residues. *Soil Science Society of America Journal*.

[36] Martinez-Trinidad S., Cotler H., Cruz-Cárdenas G. (2012). The aggregates stability indicator <ASItest> to evaluate soil spatiotemporal change in a tropical dry ecosystem. *Journal of Soil Science and Plant Nutrition*.

[37] Sung C. T. B. (2012). Aggregate stability of tropical soils in relation to their organic matter constituents and other soil properties. *Pertanika Journal of Tropical Agricultural Science*.

[38] Li Y., Ma Z., Liu Y. (2023). Variation in soil aggregate stability due to land use changes from alpine grassland in a high-altitude watershed. *Land*.

[39] Razafimbelo T. (2005). Soil carbon storage and protection in no-tillage systems of a tropical soil in malagasy highlands.

[40] Getachew F., Abdulkadir A., Lemenih M., Fetene A. (2012). Effects of different land uses on soil physical and chemical properties in Wondo Genet area. *Ethiopia. New York Science Journal*.

[41] Koull N., Halilat M. T. (2016). Effect of organic matter in physical and chemical of sandy soil properties of Ouargla region (Algeria). *Etude et Gestion des Sols*.

[42] van Leeuwen J. P., Lehtinen T., Lair G. J. (2015). An ecosystem approach to assess soil quality in organically and conventionally managed farms in Iceland and Austria. *Newton J. S., de Ruiter P. C. Soil*.

[43] Désiré T., Oumarou Y. (2016). Properties, classification, genesis and agricultural suitability of soils in a semiarid pediplain of North Cameroon. *African Journal of Agricultural Research*.

[44] Tabi F. O., Bitondo D., Yinda G. S., Kengmegne S. S. A., Ngoucheme M. (2013). Effect of long term integrated soil fertility management by local farmers on nutrient status of a Typic Dystrandept under potato-based cropping system. *International Research Journal of Agricultural Science and Soil Science*.

[45] Were O. K., Singh R. B., Dick B. O. (2015). *Effects of Land Cover Changes on Soil Organic Carbon to Advance Food Security and Enhance Climate Resilience in Africa*.

[46] Soltner D. (2005). The basics of plant production. *The soil and its improvement*.

[47] Andrist-Rangel Y., Simonsson M., Andersson S., Öborn I., Hillier S. (2006). Mineralogical budgeting of potassium in soil: a basis for understanding standard measures of reserve potassium. *Journal of Plant Nutrition and Soil Science*.

[48] Kayser M., Benke M., Isselstein J. (2012). Potassium leaching following silage maize on a productive sandy soil. *Plant Soil and Environment*.

[49] Tsozué D., Nghonda J. P., Mekem D. L. (2015). Impact of land management system on crop yields and soil fertility in Cameroon. *Solid Earth*.

[50] Dengi̇z O., Demi̇rkaya S. (2022). Estimation and spatial distribution of some soil erodibility parameters in soils of Ilgaz National Park. *Eurasian Journal of Soil Science*.

[51] Horneck A. D., Sullivan M. D., Owen S. J., Hart M. J. (2011). Soil test interpretation guide.

[52] Tripolskaja L., Kazlauskaite-Jadzevice A., Razukas A. (2023). Organic carbon, nitrogen accumulation and nitrogen leaching as affected by legume crop residues on sandy loam in the eastern baltic region. *Plants*.

[53] Doucet R. (2006). *Climate and Agricultural Soils*.

[54] Neina D. (2019). The role of soil pH in plant nutrition and soil remediation. *Applied and Environmental Soil Science*.

[55] Pypers P., Sanginga J. M., Kasereka B., Walangululu M., Vanlauwe B. (2011). Increased productivity through integrated soil fertility management in cassava–legume intercropping systems in the highlands of Sud-Kivu, DR Congo. *Field Crops Research*.

[56] Delaunois A., Ferrie Y., Bouche M., Colin C., Rionde C. (2008). Guide for the description and evaluation of soil fertility. *Chamber of Agriculture 81*.

[57] Yang M., Zhou D., Hang H. (2024). Effects of balancing exchangeable cations Ca, Mg, and K on the growth of tomato seedlings (Solanum lycopersicum L.) based on increased soil cation exchange capacity. *Agronomy*.

[58] Akpan-Idiok A. U., Ofem K. I. (2014). Physicochemical characteristics, degradation rate and vulnerability potential of obudu cattle ranch soils in southeast Nigeria. *Open Journal of Soil Science*.

[59] Fao (2005). *Soil Fertility Management for Food Security in Sub-saharan Africa*.

[60] Mulaji C. K. (2011). Use of household bio-waste composts to improve the fertility of acid soils in the Province of Kinshasa (Dem. Rep. of Congo).

[61] Nanganoa L. T., Ngome F. A., Suh C., Basga S. D. (2020). Assessing soil nutrients variability and adequacy for the cultivation of maize, cassava, and sorghum in selected agroecological zones of Cameroon. *International journal of Agronomy*.

[62] Du J., Liu K., Huang J. (2022). Organic carbon distribution and soil aggregate stability in response to long-term phosphorus addition in different land-use types. *Soil and Tillage Research*.

